# Symptomatic hyperprolactinemia mimicking pituitary pathology in a child with Landau–Kleffner syndrome, autism spectrum disorder, and intellectual disability: a case report

**DOI:** 10.1186/s13256-026-06181-x

**Published:** 2026-06-22

**Authors:** Swetang J. Shah, Viral Goradia, Aahna V. Dattani, Sireesha Palkamsetti, Usha Ravi, Anshuman Srivastava

**Affiliations:** 1 Bay Area Community Health, Fremont, CA USA; 2 Upstate Medical University, Syracuse, NY USA; 3 Valley Children’s Hospital, Fresno, CA USA; 4 Tulare Pediatric Group, Tulare, CA USA; 5 Family Health Care Network, Visalia, CA USA

**Keywords:** Landau–Kleffner syndrome, Autism spectrum disorder, Risperidone, Hyperprolactinemia, Olanzapine, Paediatric psychopharmacology, Intellectual disability, Case report

## Abstract

**Introduction:**

Symptomatic hyperprolactinemia is rarely seen in children, particularly among those with complex neurodevelopmental disorders. While risperidone is known to raise prolactin levels, cases with clear clinical symptoms are uncommon. This report describes an unusual presentation of hyperprolactinemia in a 12-year-old girl with Landau–Kleffner syndrome, autism spectrum disorder, and intellectual disability, whose symptoms closely resembled pituitary pathology.

**Case presentation:**

The 12-year-old Hispanic Peruvian female was under long-term behavioral control therapy of risperidone. With time, she got galactorrhea, visual impairments, and falls. Laboratory revealed high serum prolactin (40.2 ng/mL) and normal thyroid and negative beta-hCG level. No pituitary lesion was seen on neuroimaging. Behavioral symptoms increased following the discontinuation of risperidone, after which the substitution of the latter with olanzapine led to complete restoration. Three months later galactorrhea disappeared, vision improved, and prolactin level was restored (2.2 ng/mL). The mother of the patient was relieved to find out that it was a medication-related issue, but not a tumor and indicated a significant enhancement in the overall behavior and quality of life of her child.

**Conclusions:**

The case shows the significance of following prolactin levels in children who are on risperidone therapy particularly those who are non-verbal or those who cannot communicate any discomfort. Clinicians are to remember that risperidone leads to hyperprolactinemia which can simulate pituitary malfunctions. Early identification, educating caregivers, and substitution of prolactin-stimulating drugs with prolactin-sparing ones like olanzapine will allow avoiding unnecessary investigations and enhancing the clinical and family outcomes.

## Introduction

Landau–Kleffner syndrome (LKS) is an epileptic encephalopathy in childhood with the development of a language regression, functional limitations and is frequently associated with autism spectrum disorder (ASD) and intellectual disability (ID) [1, 2]. Risperidone, a very common antipsychotic is also prescribed to treat these children to deal with aggression and self-harming behavior [3]. Risperidone is strongly associated with elevated serum prolactin and most of the children are not even symptomatic [4]. Symptomatic hyperprolactinemia that takes the form of galactorrhea, menstrual abnormalities, or pubertal retardation is also uncommon and imitation of pituitary pathology is even more infrequent [5, 6]. Up to the present, no case of symptomatic hyperprolactinemia in a child with LKS and ASD and ID co-occurrence has been described. The diagnostic problem is exaggerated in nonverbal children, whereby there is a lack of communication that slows the realization of the side effects of the drugs. The case thus offers new clinical understanding on the overlap between rare neurodevelopmental conditions, abnormal use of antipsychotics, and endocrine complications.

## Case presentation

### Patient information

The patient is a 12-year-old Hispanic Peruvian female who presented with a complicated neurodevelopmental profile. She was delivered at 37 weeks via caesarean section due to concerns of a nuchal cord (umbilical knot) and possible intrauterine growth restriction. No birth history was notable, and no recorded perinatal complication was reported. Her early development seemed to proceed according to expectancies. She sat independently at the age of 8 months, started walking at 14 months and uttered her first words at 12 months.

Her parents noted regression of development at age about 4 years and 6 months (54 months). She started to show chronic crying, a speech loss that occurred previously, and new behavioral problems such as poor social interaction and inability to regulate emotions. Electroencephalography was abnormal but records were unavailable and was included in the neurological assessment in Peru. Initial evaluations in Peru included neuroimaging and genetic testing, which were inconclusive. She received a trial of IVIG for suspected autoimmune encephalitis without sustained benefit.

Genetic testing was performed in Peru, including chromosomal microarray and exome sequencing, though these were older techniques and yielded no definitive diagnosis. As her regression progressed, features consistent with autism spectrum disorder (ASD) became more prominent, and her overall functioning corresponded with a moderate intellectual disability (ID). Impulsivity and behavioral dysregulation were prominent, and over time she developed a phenotype consistent with overlapping traits of attention deficit hyperactivity disorder (ADHD).

In August 2022, the family immigrated to the United States, where the patient established care with a multidisciplinary team. This included neurology, psychiatry, developmental pediatrics, and specialized educational and therapeutic services at a tertiary care center.

### Diagnostic assessment

**Fig. 1:**
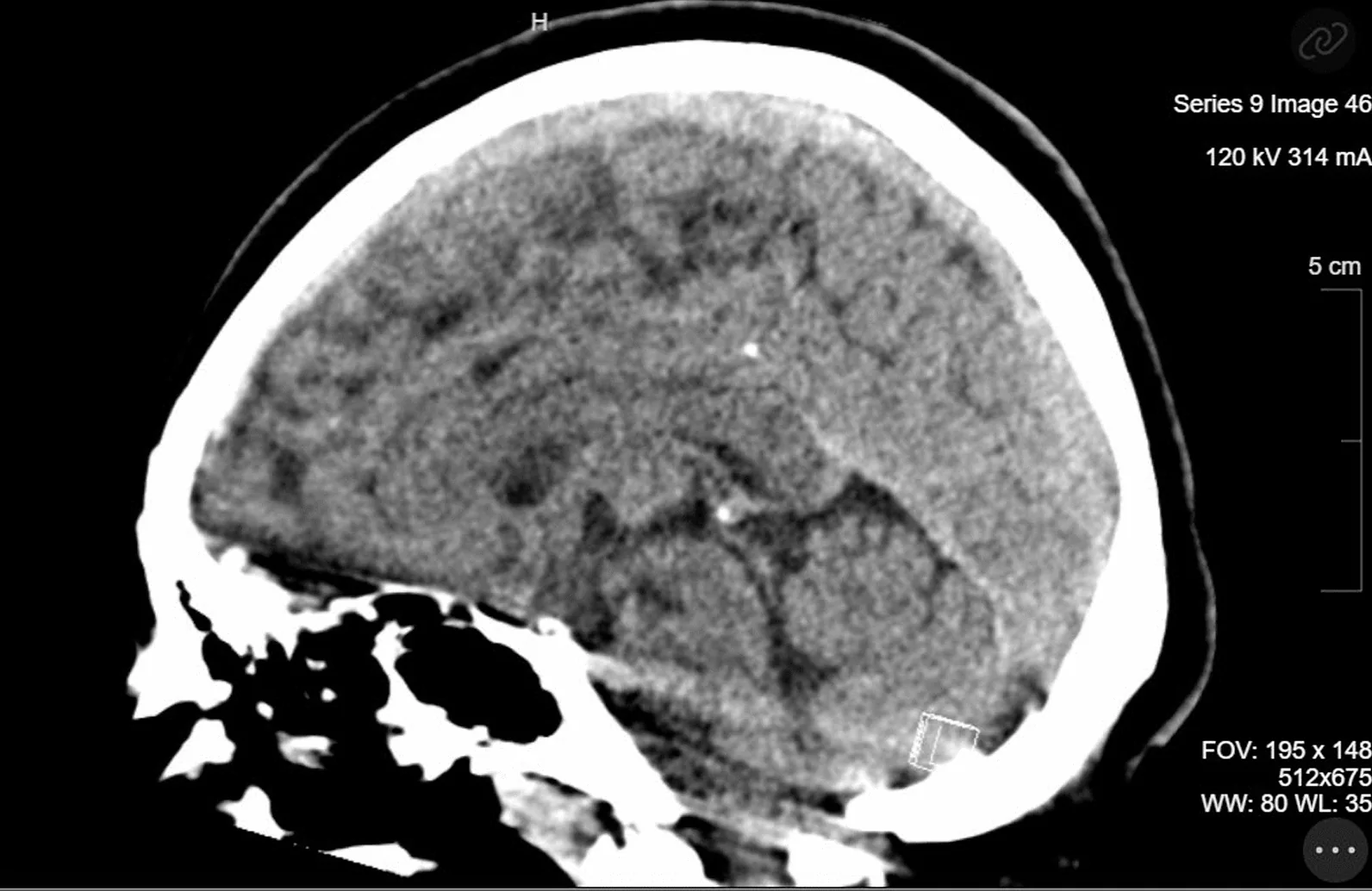
Sagittal CT brain (non-contrast) demonstrating no evidence of pituitary adenoma, mass lesion, or optic chiasmal compression

Laboratory investigations revealed an elevated serum prolactin level of 40.2 ng/mL, while thyroid function and beta-hCG tests were normal. Liver function tests were within reference ranges. Given the visual symptoms and fall episodes, neuroimaging was performed to exclude a pituitary or chiasmatic mass. Computed tomography of the brain showed no pituitary enlargement or mass effect (see Fig. 1).

**Fig. 2:**
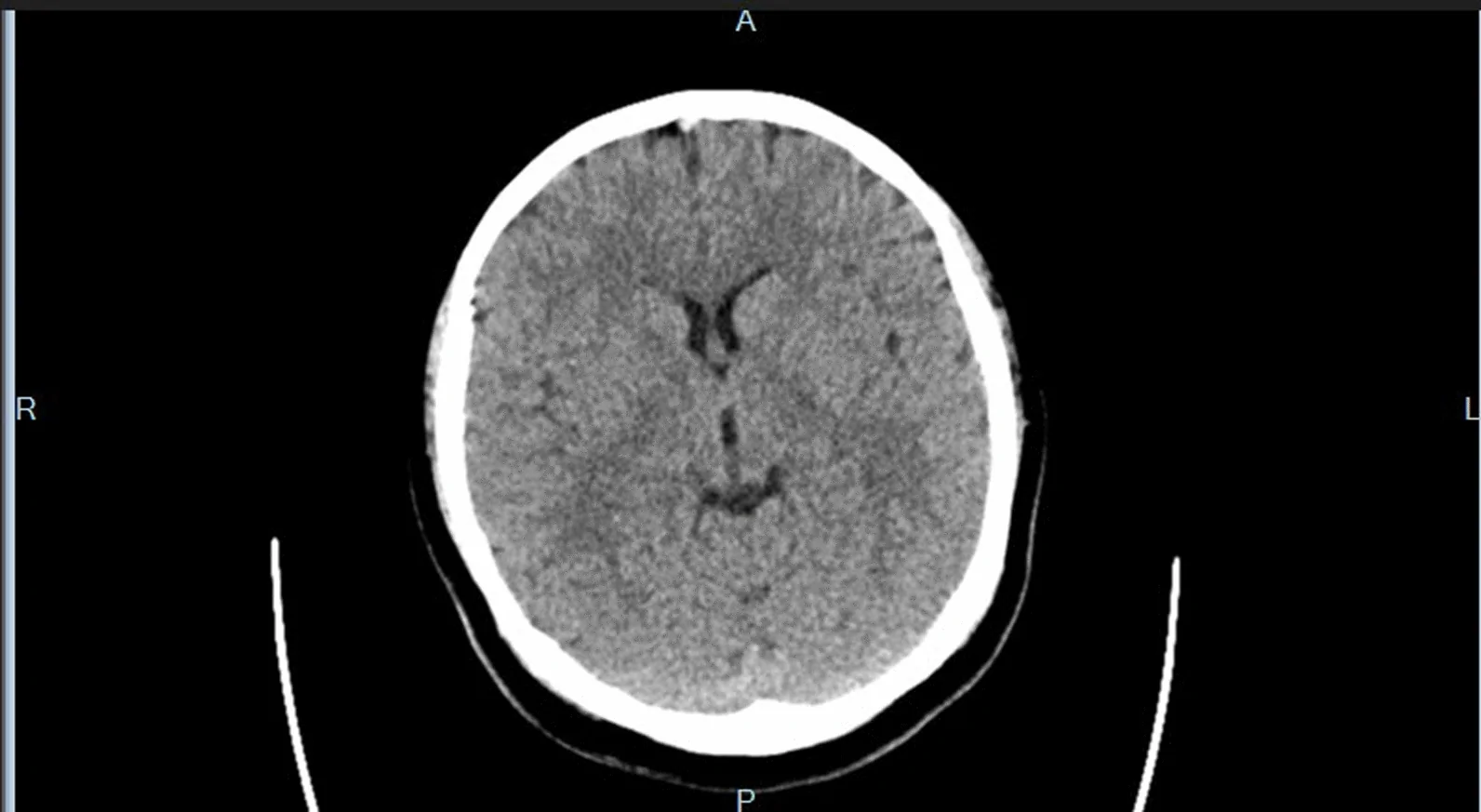
Coronal CT brain (non-contrast) showing no pituitary enlargement, mass effect, or suprasellar lesion suggestive of structural pathology

Subsequent hormone testing, after the medication switch, showed normal follicle-stimulating hormone and luteinizing hormone values. These findings support a diagnosis of risperidone-induced hyperprolactinemia rather than a structural pituitary disorder (see Fig. 2, Table 1).

**Table 1:** Laboratory findings before and after medication switch, demonstrating resolution of hyperprolactinemia following discontinuation of risperidone

Laboratory parameter	31 March 2025 (on risperidone)	11 June 2025 (post-switch to olanzapine)	Reference range*
Prolactin (ng/mL)	40.2 ↑	2.2 (normalized)	4.0–20.0
Thyroid stimulating hormone (TSH, µIU/mL)	1.33	1.0	0.5–4.5
Follicle-stimulating hormone (FSH, mIU/mL)	Not tested	5.0	1.5–12.0 (prepubertal/pubertal range)**
Luteinizing hormone (LH, mIU/mL)	Not tested	4.6	0.1–8.0 (prepubertal/pubertal range)**
Beta-hCG, qualitative	Negative	Not tested	Negative
Liver function tests	Normal	Normal	Within reference limits

^*^Reference ranges are provided as general pediatric laboratory intervals; ranges may vary slightly between laboratories

^**^Pubertal reference ranges depend on Tanner stage. Reported values for FSH and LH were within expected limits for a 12 years (144 months)

↑ = elevated relative to reference range

### Therapeutic interventions

Risperidone therapy was discontinued in April 2025 once the elevated prolactin level and galactorrhea were identified. Shortly after withdrawal, the patient exhibited worsening aggression, impulsivity, and self-injurious behaviors, creating considerable strain on her caregivers. Trials of fluoxetine and escitalopram were attempted for behavioral control but were stopped because of inadequate response and adverse effects. A psychiatric review led to the introduction of olanzapine at 2.5 mg four times daily. The drug was well-tolerated, and within 4 weeks, the galactorrhea resolved completely, and behavioral stability returned.

### Follow-up and outcomes

**Fig. 3:**
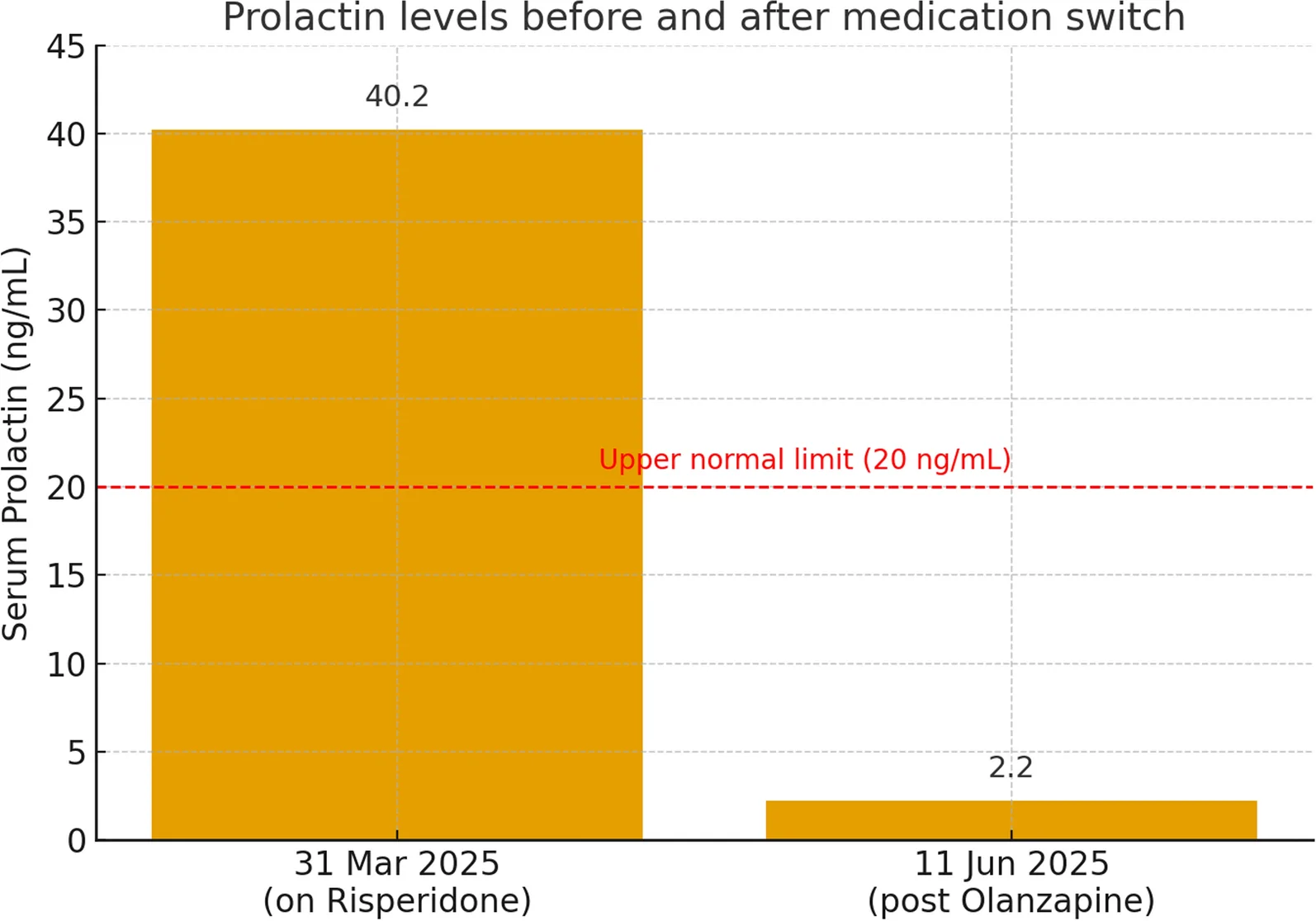
Serum prolactin levels before and after discontinuation of risperidone and initiation of olanzapine, demonstrating normalization following medication change

By June 2025, repeat laboratory tests showed a normalized prolactin concentration of 2.2 ng/mL and stable thyroid and gonadotropin levels (see Fig. 3).

The patient’s vision improved, and her gait steadied. Her mother reported substantial relief after learning that the symptoms were drug related rather than due to a tumor. She also described better quality of life for both her and her daughter once olanzapine achieved behavioral control. No further episodes of galactorrhea or vision problems were noted in the months that followed, and the child continued to attend special education sessions and regular medical reviews without complications.

## Discussion

This case presents the clinical and treatment challenge of the diagnosis and treatment of the hyperprolactinemia caused by antipsychotics in a child having a rare and complex neurodevelopmental presentation. The case presented of the patient reveals how comorbid neurological and psychiatric disorders, together with the weak communication skills, can mask the sign of drug side effects.

### Diagnostic challenge

The emergence of galactorrhea in a 12-year-old girl on long-term risperidone was accompanied by vision loss, clumsiness, and frequent falls. These symptoms initially raised concern for a pituitary mass compressing the optic chiasm, a possibility that required urgent exclusion. Computed tomography demonstrated no evidence of a mass lesion, and the markedly elevated prolactin level of 40.2 ng/mL, in the absence of other endocrine abnormalities, pointed to a drug-induced mechanism. The clinical dilemma lay in distinguishing between structural pathology and medication-related endocrine disturbance, a challenge amplified by the patient’s inability to reliably report symptoms due to aphasia and intellectual disability. In such cases, the threshold for pursuing imaging remains low, as structural pathology cannot be excluded on history and physical examination alone.

### Review of similar published cases or literature

The case aligns with existing literature demonstrating that risperidone is frequently associated with elevated prolactin levels in pediatric populations [7, 8]. Hellings et al. [4] and Anderson et al. [2] reported high rates of biochemical hyperprolactinemia, although most cases were asymptomatic. While clinically significant manifestations such as galactorrhea have been described in selected cases, visual disturbances have not been clearly reported in the context of antipsychotic-induced hyperprolactinemia. This distinction is clinically important, as visual symptoms typically prompt evaluation for structural pituitary pathology. The present case, therefore, adds to the literature by describing a unique presentation in which drug-induced hyperprolactinemia mimicked features suggestive of a pituitary lesion, including visual symptoms that were fully reversible following medication adjustment.

This case illustrates that children with complex neurodevelopmental disorders are at heightened risk of delayed recognition of adverse drug reactions. Routine prolactin monitoring, caregiver education, and timely medication adjustments are critical safeguards. The key take-away is that risperidone-induced hyperprolactinemia may closely mimic pituitary pathology, and switching to a prolactin-sparing antipsychotic such as olanzapine can restore both endocrine and behavioral stability (see Table 2).

**Table 2:** Clinical timeline highlighting onset of symptoms, diagnostic evaluation, and resolution following medication change

Approximate date/age	Clinical event
Birth (37 weeks)	Delivered via C-section in Peru; knot in umbilical cord, no major perinatal complications
8 months	Sat independently
14 months	Walked independently
12 months	Spoke first words
~ 4.5 years	Regression of speech and social interaction; diagnosed with Landau–Kleffner syndrome
~ 5 years	Risperidone initiated for aggression and self-injury
Mid-2022 (Age ~ 9 years)	Family migrated to the United States; multidisciplinary care established
Early 2025 (Age 12 years)	Developed galactorrhea, visual disturbance, and frequent falls; elevated prolactin detected
Mid-2025	Risperidone discontinued → noticeable behavioral relapse
Mid-2025	Switched to olanzapine → galactorrhea resolved; prolactin normalized; behavioral stabilization achieved

### Clinical reasoning for diagnosis and management

The patient presented with a combination of galactorrhea, progressive vision changes, and frequent falls, which at first suggested a possible pituitary or chiasmatic mass. However, the absence of other neurological deficits, normal thyroid and liver function, and the known association between risperidone and elevated prolactin levels directed clinical reasoning toward a medication-induced cause. The markedly raised serum prolactin, in the context of normal beta-hCG and unremarkable imaging, confirmed the diagnosis of drug-induced hyperprolactinemia rather than structural pituitary disease.

Management decisions balanced two competing priorities: resolving the endocrine side effects and maintaining behavioral stability in a child with multiple neurodevelopmental disorders. Simply withdrawing risperidone relieved the galactorrhea but led to severe behavioral relapse, which highlighted the need for a replacement therapy with a safer hormonal profile. Olanzapine was selected for its lower tendency to increase prolactin while retaining antipsychotic efficacy [9]. The rapid normalization of prolactin and improvement in behavior after the medication switch validated this approach. Regular laboratory monitoring and close caregiver communication were key in maintaining both safety and stability.

### What this case adds to existing knowledge

Although risperidone-related hyperprolactinemia is well-documented in children, most cases remain asymptomatic. This report contributes a rare example of a symptomatic presentation in a young girl with combined Landau–Kleffner syndrome, autism spectrum disorder, and intellectual disability. It demonstrates that such children are particularly vulnerable to delayed recognition of adverse effects because of limited communication and overlapping behavioral symptoms. The case also supports the effectiveness of olanzapine as an alternative when prolactin elevation causes clinical symptoms, showing that it can restore endocrine balance without worsening psychiatric outcomes. By integrating the caregiver’s perspective, this report further emphasizes the psychosocial impact of delayed recognition and highlights the importance of multidisciplinary management.

### Proposed pathophysiological mechanism

Functional and biochemical impacts of hyperprolactinemia may explain the existence of visual disturbance and gait instability in this patient, although no structural pituitary lesion was detected. Dopamine D2 receptor blockage by risperidone causes persistent increase of prolactin which can have an indirect effect on hypothalamic–pituitary communication and change neuroendocrine balance. Higher levels of prolactin have been linked to fluid retention and mild cases of pituitary enlargement, which may temporarily impair the optic chiasm, but are not a full-blown adenoma. One may also consider that neurobehavioral factors or effects of medications, or disturbed sensory processing as a result of underlying neurodevelopmental disorders, may have contributed to visual symptoms. A reversible and drug induced pathophysiologic process and not structural pathology is supported by the fact that the entire symptoms are resolved after cessation of risperidone use.

### Key clinical lessons or red flags for practitioners

1. Regularly check the levels of prolactin in children on chronic risperidone, even without the symptoms.
2. Keep an eye on minor red flags like new discharge of the breast, visual disturbances or unsteady walking which could be signs of serious hyperprolactinemia.
3. Eliminate structural pathology by focusing on medication side effects, imaging must be done to be sure there is no pituitary lesion before assigning the symptoms to solely the drug.
4. Train caregivers to identify early hormonal or behavioural changes, which they frequently identify with subtle problems before they visit clinical offices.
5. Antipsychotics, including olanzapine or aripiprazole, should be considered, taking into consideration the appearance of the symptoms, to balance behavioral control with the endocrine system.

Overall, the case can serve as a reminder to clinicians that even common drugs may create some unforeseen endocrine issues and that caring, attentive, and interdisciplinary treatment can help to avoid painful experiences of both the child and the family.

## Limitations

This report is limited by its single-case design and the absence of advanced imaging, such as MRI, which may have provided greater sensitivity in detecting subtle pituitary changes. In addition, objective ophthalmologic assessment of visual symptoms was limited.

## Conclusion

The current case is considered to be an unusual and complex situation of symptomatic hyperprolactinemia in a long-term risperidone-treated child with the Landau–Kleffner syndrome, autism spectrum disorder, and intellectual disability. The diagnostic dilemma of distinguishing between endocrine effects caused by drugs and structural pathology in nonverbal children was emphasized by clinically significant galactorrhea, complaints of clumsiness and vision developed in the patient that was highly reminiscent of a pituitary lesion.

Resolution of hyperprolactinemia following discontinuation of risperidone, along with restoration of behavioral stability after initiation of olanzapine, underscores the importance of considering prolactin-sparing alternatives in cases of symptomatic adverse effects. Another issue which is outlined in the case is the risk of the inability to identify adverse drug reactions promptly, because their manifestation might be considered by the caregivers and clinicians as a comorbidity to the behavioral or neurological symptoms, which have already been present.

Regular prolactin checks, active education of the caregivers and strict multidisciplinary cooperation are important to protect the outcomes in the children who might not be able to reliably describe the symptoms. In spite of the fact that risperidone is still commonly employed in the treatment of aggression and irritability in neurodevelopmental disorders in children, a closer focus on endocrine outcomes is required, especially in the nonverbal and vulnerable groups in this report. Further investigation is required in order to develop a set of definite rules regarding the management of hyperprolactinemia caused by antipsychotics in children, its future discontinuation or reduction, and change of treatment.

## Data Availability

All data generated or analyzed during this study are included in this published article.
